# Prevalence of Posttraumatic Stress Symptoms and Associated Characteristics Among Patients With Chronic Pain Conditions in a Norwegian University Hospital Outpatient Pain Clinic

**DOI:** 10.3389/fpsyg.2020.00749

**Published:** 2020-05-05

**Authors:** Lene Therese Bergerud Linnemørken, Lars-Petter Granan, Silje Endresen Reme

**Affiliations:** ^1^Division of Emergencies and Critical Care, Department of Research and Development, Oslo University Hospital, Oslo, Norway; ^2^Division of Emergencies and Critical Care, Department of Pain Management and Research, Oslo University Hospital, Oslo, Norway; ^3^Department of Psychology, Faculty of Social Sciences, University of Oslo, Oslo, Norway

**Keywords:** chronic pain, comorbidity, posttraumatic stress disorder, trauma, psychological factors

## Abstract

**Introduction:**

Comorbid posttraumatic stress disorder (PTSD) in patients with chronic pain may have a negative effect on the course and outcome of both disorders. Nevertheless, the co-occurrence of the two conditions is often overlooked in clinical settings. Further, little is known about how PTSD is associated with biopsychosocial characteristics in this patient group. The first objective was to assess the prevalence of posttraumatic stress symptoms (PTSS) in patients with chronic pain in a Norwegian university hospital outpatient pain clinic. The second objective was to investigate possible associations between PTSS and adverse outcomes such as pain intensity, disability, and distress. The third objective was to compare the PTSS prevalence rates between primary versus secondary pain conditions.

**Materials and methods:**

Six hundred and ninety-two patients meeting for pain assessment completed self-report questionnaires about PTSS and possibly associated factors. The Life Events Checklist and the Stressful Life Events Screening Questionnaire were used to screen for potentially traumatic life events. The Impact of Events Scale – Revised and the PTSD Checklist for DSM-5 were used to assess PTSS. Differences between patients with and without severe PTSS on the possibly associated variables were analyzed by chi-squared-, and *t*-tests.

**Results:**

20.7% of the participants reported a level of PTSS qualifying for a PTSD diagnosis. These patients reported higher levels of pain intensity, pain bothersomeness, disability, and psychological distress, as well as lower levels of self-efficacy. They also reported higher levels of pain catastrophizing, perceived injustice, fatigue, and sleep difficulties. Finally, there was not a significant difference in prevalence rates between primary and secondary pain conditions.

**Discussion:**

PTSS are frequent in patients with chronic pain, and a range of psychological characteristics is associated with a high level of such symptoms in this patient group. Patients with both conditions report a significantly higher symptom load, and the potential impact on the individual’s life is major. In terms of pain condition, there were no differences in PTSS between primary pain conditions and secondary pain conditions in this pain population. This study emphasizes the importance of increased attention on PTSS when seeing patients with chronic pain conditions in clinical practice.

## Introduction

The importance of comorbid physical and psychological disorders in patients with chronic pain conditions has been emphasized in the last half-century, as the understanding of pain as a multidimensional experience has developed. A relationship between psychiatric disorders such as depression and anxiety and chronic pain was early suggested (Banks and Kerns, 1996; Asmundson et al., 1996), while in the later years the association between chronic pain and posttraumatic stress disorder (PTSD) has gained increased attention. PTSD is classified as a stress-related disorder in DSM-5 (American Psychiatric Association, 2013), and the condition may develop after exposure to actual or threatened death, serious injury, or sexual violence. PTSD is characterized by re-experiences, avoidance, negative thoughts or feelings, and trauma-related arousal or reactivity (American Psychiatric Association, 2013). The condition is associated with an increased risk of psychological and physical health problems, and individuals suffering from PTSD often display high levels of physical, occupational, and social disability (American Psychiatric Association, 2013). Pain is among the physical symptoms most frequently reported by individuals with PTSD, and it is found independent of the nature of the trauma (Asmundson et al., 2002).

The first step to gain insight into the consequences of PTSD in patients with chronic pain conditions is to examine how frequently the two conditions co-occur. In a recent systematic review, 16 of 19 studies were found to demonstrate an association between the two conditions (Fishbain et al., 2017). The rates of this co-occurrences reported are, however, widely varying. A meta-analysis of PTSD prevalence among individuals with chronic pain conditions published in 2017 (Siqveland et al., 2017) found a variation in PTSD rates from 0 to 57%, and a pooled variance of 9.7%. Also, different rates of PTSD have been found depending on the patient group in question, with the lowest prevalence rate (0.69%) found among patients suffering from chronic low back pain, and the highest rate (50.1%) found in veterans (Fishbain et al., 2017). PTSD prevalence may also vary considerably between different pain conditions depending on the temporal relationship between the pain and the PTSD (Fishbain et al., 2017). As soldiers are more likely to develop PTSD after experiencing combat events if they get physical injuries during the event (which may also cause chronic pain; Koren et al., 2005), the comorbidity between chronic pain and PTSD may be more frequently seen when both conditions are caused by the same event. Variation in observed rates may also be caused by a lack of standardized measurement of either condition (Beckham et al., 1997), which emphasize the need for studies with more rigorous assessments of both PTSD and the pain condition(s).

As far as we know, only two previous studies have examined the prevalence of PTSS in patients with chronic pain conditions consecutively accepted for pain rehabilitation in Scandinavia. In the first study, 23% of patients assessed at hospital pain clinics in Finland and Denmark reported PTSS at a level qualifying for a PTSD diagnosis (Andersen et al., 2012). In the second study, 29% of patients meeting for pain assessment at a Swedish hospital pain clinic screened positive for PTSD (Akerblom et al., 2017). Despite these high rates, the comorbidity of chronic pain and PTSS often goes unrecognized in clinical settings.

The importance of correctly identifying patients with chronic pain and PTSS/PTSD in clinical practice is evident, as PTSS may be associated with a range of physical and psychological symptoms (Beckham et al., 1997; Palyo and Beck, 2005; Russek et al., 2015). Further, preliminary research suggest that such comorbidity may have a significant effect on the course and outcome for either condition (Otis et al., 2003). In the aforementioned Swedish study, patients with both conditions reported significantly higher levels of pain interference, kinesiophobia, anxiety and depression, as well as significantly lower levels of life control (Akerblom et al., 2017). There is, however, a wide range of factors possibly associated with severe PTSS in patients with chronic pain that has not yet been investigated. For example, very few studies have examined the role of pain catastrophizing, and the possible association between high levels of PTSS and perceived injustice in this patient group. In addition, even though fatigue and sleep problems are known comorbidities in chronic pain populations (Smith and Haythornthwaite, 2004; Creavin et al., 2010), very little is known about their association with PTSS in patients with chronic pain. As targeting factors associated with PTSS in clinical settings may be the key to a better treatment outcome for this patient group, the need for further investigation is evident.

The main objective of this study was to assess the prevalence of PTSS in patients with chronic pain conditions consecutively accepted for assessment at a Norwegian university hospital outpatient pain clinic. The secondary objective was to examine whether high levels of PTSS are associated with adverse outcomes, including pain intensity, pain bothersomeness, level of disability, degree of affective distress, pain catastrophizing, self-efficacy, perceived injustice, fatigue, and sleep difficulties in patients with chronic pain conditions. Finally, the third objective was to investigate whether PTSS prevalence varied between primary and secondary pain conditions.

## Materials and Methods

### Participants

Patients consecutively assessed at the largest university and interdisciplinary outpatient pain clinic in Norway were invited to participate in the study. Data collection was administered in two waves, (1) from May to July 2016, and (2) from March to December 2019. Patients from all parts of Norway are referred to the clinic, and the conditions presented are often complex. Pain assessment and treatment are provided by a team of specialists, including physicians, clinical psychologists, physiotherapists, nurses, and an occupational therapist. All patients meeting for their first consultation at the clinic were asked to participate in the study. It is standard procedure at the clinic that all new patients complete a package of self-reported questionnaires on a tablet before their first appointment. Patient data collected are included in a local quality registry. The additional questionnaires necessary for the current study (LEC, SLESQ, IES-R, and PCL-5) were added to the standard package in the given time periods. Exclusion criteria was age younger than 18 or insufficient Norwegian language abilities.

### Ethical Aspects

The Data Protection Officer at Oslo University Hospital approved the research protocol for the local quality registry at the pain clinic (ref. 2014/1309). All principles in the Helsinki Declaration were followed. Written consents were obtained before any study-related procedure took place. The right to withdraw from the study at any time without any explanation and without consequences for follow-up and treatment at the clinic was emphasized. If patients reported a level of PTSS considered to be especially high (a score of ≥ 45 on IES-R/PCL-5; 12 points or more above the chosen cut off for screening positive for PTSD), his or her physician and/or psychologist at the clinic was informed the same day.

### Outcome Measures

In the first wave of the data collection, we measured exposure to potentially traumatic life events by use of a validated Norwegian version of the Life Events Checklist (LEC) (Blake et al., 1995; Halvorsen and Stenmark, 2010). The psychometric properties of LEC are found to be good (Elhai et al., 2005), and it converges well with other established measures of exposure to potentially traumatic events (Gray et al., 2004). The LEC is a 17-item questionnaire, and patients were asked to indicate whether they had been exposed to each listed event during their lifetime by selecting one of the following options: 1 = ‘It happened to me’, 2 = ‘Has witnessed it’, 3 = ‘Has learned it has happened to someone close’, 4 = ‘Not sure’, and 5 = ‘Does not apply to me’. Multiple answers were allowed. If the patient selected one of the three first response options on one or more items, the A Criterion for PTSD in DSM-IV (American Psychiatric Association, 1994) was considered fulfilled.

In the second wave of the data collection, the Stressful Life Events Screening Questionnaire (SLESQ) (Goodman et al., 1998) was used to measure exposure to potentially traumatic life events. A Norwegian translation was used (Thoresen and Øverlien, 2013). SLESQ has been found to demonstrate satisfactory validity and a good ability to discriminate between events categorized as Category A events and those events that are not (Goodman et al., 1998). SLESQ consists of 15 items, in which each item represents a potentially traumatic life event. Patients were asked to indicate the presence or absence of exposure to each of the 15 events listed, by choosing between the options “Yes” or “No”.

Patients who confirmed exposure to at least one potentially traumatic life event were asked to complete questionnaires regarding PTSS. In the first wave of the data collection, PTSS were measured using the Impact of Events Scale – Revised (IES-R) (Weiss and Marmar, 1997). A Norwegian version of IES-R with satisfactory reliability and accuracy was used (Eid et al., 2009). IES-R assesses PTSS experienced in the last week, and it corresponds to the symptoms of PTSD listed in DSM-IV (American Psychiatric Association, 1994). IES-R has 22 items, and patients report how often they experience each symptom by selecting an option on a 4-point Likert scale (1 = ‘Not at all’ to 4 = ‘Very often’). Scores on all items were then summed, and a score of ≥ 33 indicated a symptom load qualifying for a PTSD diagnosis (Creamer et al., 2003).

In the second wave of the data collection, symptoms of posttraumatic stress were measured using a Norwegian translation of the PTSD Checklist for DSM-5 (PCL-5) (Heir, 2013; Weathers et al., 2013). PCL-5 is a widely used self-report measure for assessment of PTSS experienced during the last month, and it has demonstrated strong reliability and validity (Blevins et al., 2015). The 20 items in the questionnaire correspond to each of the PTSD symptoms in DSM-5 (American Psychiatric Association, 2013), and are scored on a Likert scale ranging from 0 (‘Not at all’) to 4 (‘Extremely’). Item scores were summed, and scores above 32 were considered to represent a level of PTSS qualifying for a PTSD diagnosis (Creamer et al., 2003; Blevins et al., 2015; Bovin et al., 2016; Verhey et al., 2018).

Pain intensity and pain bothersomeness were measured using the Numeric Rating Scale (NRS). A Likert scale ranging from 0 (‘Not at all’) to 10 (‘Worst imaginable’) was used. In addition, the patients were asked to write down the duration of their pain in years and months.

Self-perceived health was measured using a 4-point Likert scale, with the answer options ‘Very good’, ‘Good’, ‘Not so good’, and ‘Poor’. This one-item scale has been widely used as an indication of subjective health status in previous studies (Idler and Benyamini, 1997; Jylha, 2009; Benyamini, 2011).

Pain-related disability was measured using a Norwegian modified version of the Oswestry Disability Index (ODI) (Baker et al., 1990; Grotle et al., 2003). ODI was originally developed to assess low back pain, so it was modified to assess pain in general by deleting the word “back” (which occurs once, in the introduction of the questionnaire). The Norwegian version of ODI has demonstrated satisfactory psychometric properties (Grotle et al., 2003). ODI consists of 10 items, and each item has six alternative response options (Likert scale, from 0 = ‘No problem performing the activity’ to 5 = ‘Pain is preventing all performance of the activity’). Scores were converted to a percentage score (range 0–100) and categorized as minimal disability (0–20%), moderate disability (21–40%), severe disability (41–60%), crippled (61–80%), or bed bound/exaggerating symptoms (81–100%).

Psychological distress was measured using the Hopkins Symptom Checklist-25 (HSCL-25) (Derogatis et al., 1974). A validated Norwegian version of the HSCL-25 with good psychometric properties (Strand et al., 2003) was applied. The 25 items concern symptoms of depression, anxiety and somatization experienced during the last week, and patients were asked to indicate degree of distress caused by each item on a 4-point Likert scale (from ‘Not at all’ to ‘Extremely’). Total scores and subscale scores were calculated by averaging item scores (range 1–4). Recommended cut-off score is 1.67 for men and 1.75 for women, and scores above the cut-off value suggest a symptom load consistent with a psychiatric disorder (Sandanger et al., 1998).

Pain catastrophizing was measured using the Pain Catastrophizing Scale (PCS) (Sullivan et al., 1995). A Norwegian translation with high reliability and validity (Fernandes et al., 2012) was applied. The PCS has 13 items related to pain perception, and patients were asked to reflect on past painful experiences when completing the questionnaire. Each item has five answer options (Likert scale, from 0 = ‘Not at all’ to 5 = ‘All the time’), and item scores were summarized to provide a PCS total score (range 0–52). A total score of ≥ 30 has been found to have clinical relevance (Sullivan, 2008a).

Self-efficacy was measured using a Norwegian translation of the General Perceived Self-Efficacy Scale (GSE) (Schwarzer and Jerusalem, 1995; Røysamb et al., 1998). The good psychometric properties of the GSE has been well supported (Nilsson et al., 2015). The GSE comprises 10 items, and patients were asked to indicate the degree to which they agree with each item on a general basis (Likert scale, from 0 = ‘Not true at all’ to 4 = ‘Exactly true’). All items were summed to provide a total score (range 10–40). Higher scores indicated higher levels of self-efficacy.

Experience of injustice was measured using the Injustice Experience Questionnaire (IEQ) (Sullivan, 2008b). A Norwegian version approved by the authors (Ljoså et al., 2015) was used. IEQ is a 12-item measure, and patients were asked to indicate how often they experienced thoughts and feelings of injustice on a 5-point Likert scale (from 0 = ‘Never’ to 4 = ‘All the time’). Item scores were summed to provide a total score (range 0 – 48). Higher scores represented higher levels of injustice, and the best cut-off value for a clinical relevance has been found to be 33 (Sullivan, 2008b).

Level of fatigue was measured using a validated Norwegian translation of the Chalder Fatigue Scale 11 (CFQ-11), also known as the Fatigue Scale (Chalder et al., 1993; Loge et al., 1998; Bjornvatn, 2016). CFQ-11 assesses the severity and extent of psychical and mental fatigue experienced in the last month, and it is widely used in epidemiological studies with both clinical and non-clinical populations. Both the reliability and validity of the scale have been found satisfactory (Fong et al., 2015). A 4-point Likert scale was used to answer each of the 11 items, and the options range from 0 (‘Better than usual’) to 3 (‘Much worse than usual’). The sum score can range from 0 to 33, but a bimodal score ranging from 0 to 11 can also be obtained. A binary score of ≥ 4 equates severe fatigue (Chalder et al., 1993).

Sleep difficulties were measured using the Insomnia Severity Inventory (ISI) (Bastien et al., 2001). ISI is a self-administered questionnaire in which patients report on the severity of insomnia experienced during the last two weeks. A Norwegian translation was used. The good psychometric properties of ISI have been demonstrated by several studies (Morin et al., 2011). The seven items on ISI were scored on a Likert scale with options ranging from 0 (‘No problem’) to 4 (‘Very severe problem’). Item scores were summed, to yield a score between 0 and 28. A total score of ≥ 15 was considered to represent a clinically significant level of insomnia (Bastien et al., 2001).

Pain diagnoses were categorized according to the classification in ICD-11 (World Heatlh Organization, 2018), which introduces a new classification system for chronic pain. ICD-11 distinguishes between chronic primary pain conditions and conditions that are associated with chronic pain (secondary pain). Chronic primary pain includes conditions that are not better explained by another diagnosis of chronic pain, for example chronic widespread pain, non-specific chronic musculoskeletal pain, primary headaches and conditions such as irritable bowel syndrome and chronic pelvic pain. Chronic secondary pain is organized into six categories; chronic cancer-related pain, chronic postsurgical or posttraumatic pain, chronic secondary musculoskeletal pain, chronic secondary visceral pain, chronic neuropathic pain, and chronic secondary headache or orofacial pain (World Health Organization, 2018).

### Statistical Analyses

SPSS version 26.0 for Mac was used for all statistical analyses. The first research question was answered by the use of descriptive statistics, while inferential statistics were used to answer the second and third research question. Groups for further analyses were defined by recoding the IES-R and the PCL-5 variables for PTSD into a binary variable (IES-R/PCL-5 total score < 33 = screening negative for PTSS at a level qualifying for a PTSD diagnosis and IES-R/PCL-5 total score ≥ 33 = screening positive for PTSS at a level qualifying for a PTSD diagnosis). Validated cut-off scores were used for categorizing patients into the two groups (Creamer et al., 2003; Blevins et al., 2015; Bovin et al., 2016; Verhey et al., 2018).

Chi-squared analyses were used to analyze group differences on categorical variables (demographic factors), and independent-sample, two-tailed, *t*-tests were used to compare the group with a high level of PTSS to the group with a low level of PTSS on continuous variables (pain intensity, pain bothersomeness, disability level, psychological distress, pain catastrophizing, perceived self-efficacy, injustice experiences, fatigue, and sleep difficulties). All assumptions for the *t*-tests were checked and met. Effect sizes were then calculated for all variables, by the use of Cramer’s V for the categorical variables and Cohen’s d for the continuous variables.

Pain diagnoses were collected from the patients’ medical records, and categorized into two groups: (1) primary pain and (2) secondary pain. Patients with secondary pain conditions were then categorized into the six categories outlined in ICD-11 (chronic cancer-related pain, chronic postsurgical or posttraumatic pain, chronic secondary musculoskeletal pain, chronic secondary visceral pain, chronic neuropathic pain, and chronic secondary headache or orofacial pain) (World Health Organization, 2018). Fisher’s exact test was used to analyze the differences in PTSS prevalence between the pain categories, as the assumptions for the chi-squared test were not met (more than 20% of the cells had expected frequencies < 5).

To investigate whether the associations between PTSS and the adverse outcomes could be spurious, adjusted analyses (multiple logistic regression) with significant demographic variables as possible confounders were performed.

## Results

### Study Sample

Eight hundred and one patients were invited to participate in the study (wave 1, *n* = 119, wave 2, *n* = 682), of which 692 consented to participate. Due to incomplete or missing data, 24 patients were excluded from further participation. Data sets were considered incomplete if <50% of the items on the questionnaires were left unanswered by the participant, and missing if no item was answered.

### Clinical Characteristics

A large majority of the participants (*n* = 538; 80.5%) reported exposure to at least one potentially traumatic event. On average, the participants had been exposed to (experienced, witnessed, or learned about it) 3.0 (*SD*: 2.95) potentially traumatic events. Life-threatening disease was the most frequently reported event on LEC (*n* = 64; 64.4%), followed by transportation accidents (*n* = 59; 58.4%), and sudden, unexpected death of someone close (*n* = 30; 54.5%). On SLESQ, the most frequently reported events were other event not listed (*n* = 191; 34.4%), life-threatening disease (*n* = 160; 28.5%) and sudden, unexpected death of someone close (*n* = 140; 24.9%). Further, more than every fifth participant (*n* = 122; 20.7%) reported a level of PTSS qualifying for a PTSD diagnosis (IES-R/PCL-5 total score ≥ 33). Descriptive data for the study population are presented in Table 1. Descriptive data for the first and the second wave of data collection are presented in Table 2 and 3, respectively.

**TABLE 1:** Chi-squared- and *t*-tests showing the differences between chronic pain patients screening positive and negative for PTSS at a level qualifying for a PTSD diagnosis in the study sample.

	**Total**		**Screening positive for PTSD**		**Screening negative for PTSD**			
			
	***n***	**%**	***n***	**%**	***n***	**%**	**t/χ^2^**	**V/d**
Gender:	576							
Male	213	37.0	40	33.3	173	37.9		
Female	363	63.0	80	66.7	283	62.1	0.87	0.04
Age:	589							
18–50	349	59.3	86	70.5	263	56.3		
≥ 51	240	40.7	36	29.5	204	43.7	8.05^∗∗^	–0.12
Diagnostic group:	588							
Primary pain	314	53.4	74	60.7	240	51.5		
Secondary pain	274	46.6	48	39.3	466	48.5	3.26	0.07
Secondary pain category:	274							
Chronic cancer-related pain	3	1.1	0	0	3	100.0		
Chronic postsurgical or posttraumatic pain	27	9.9	4	14.8	23	85.2		
Chronic secondary musculoskeletal pain	71	25.9	11	15.5	60	84.5		
Chronic secondary visceral pain	19	6.9	3	15.8	16	84.2		
Chronic neuropathic pain	149	54.4	29	19.5	120	80.5		
Chronic secondary headache or orofacial pain	5	1.8	1	20.0	4	80.0	1.10	0.07
Living situation:	561							
Alone	168	29.9	38	33.0	130	29.1		
With others	393	70.1	77	67.0	316	70.9	0.66	0.03
Marital status:	570							
Unmarried	345	60.5	84	70.6	261	57.9		
Married	225	39.5	35	29.4	190	42.1	6.37^∗^	0.11
Children:	571							
Have children	373	65.3	72	60.5	301	66.6		
No children	198	34.7	47	39.5	151	33.4	1.54	0.05
Work or life situation:	556							
Working/student/military service	215	38.7	33	28.9	182	41.2		
Not working	341	61.3	81	71.1	260	58.8	5.72^∗^	0.10

	**M**	***SD***	**M**	***SD***	**M**	**SD**	**t/*χ*^2^**	**V/d**

Age (18–92) (*n* = 589)	47.53	15.56	43.35	13.27	48.62	15.94	3.74^∗∗∗^	0.34
NRS Pain Intensity (0–10) (*n* = 551)	7.17	1.71	7.58	1.55	7.07	1.73	–2.83^∗∗^	–0.30
NRS Pain Distress (0–10) (*n* = 553)	7.78	1.68	8.19	1.49	7.68	1.71	–2.89^∗∗^	–0.31
Pain Duration in Years (0–99) (*n* = 557)	7.55	8.35	8.26	8.55	7.38	8.30	–0.99	–0.11
Self-Perceived Health (1–4) (*n* = 557)	3.16	0.73	3.47	0.55	3.09	0.74	–5.13^∗∗∗^	–0.55
The Modified ODI (0–100) (*n* = 589)	42.61	17.68	50.04	17.98	40.66	17.10	–5.34^∗∗∗^	–0.54
HSCL-25 (1–4) (*n* = 555)	2.17	0.59	2.72	0.54	2.03	0.51	–12.44^∗∗∗^	–1.32
PCS (0–52) (*n* = 555)	24.13	12.36	33.45	11.12	21.88	11.57	–9.40^∗∗∗^	–1.01
GSE (10–40) (*n* = 553)	28.62	5.73	26.35	5.86	29.18	5.56	4.71^∗∗∗^	0.50
IEQ (0–48) (*n* = 553)	23.99	11.18	31.55	9.70	22.14	10.74	–8.35^∗∗∗^	–0.89
CFQ (0–11) (*n* = 555)	7.01	3.17	8.41	2.42	6.66	3.23	–6.30^∗∗∗^	–0.57
ISI (0–28) (*n* = 556)	15.42	6.83	19.30	6.51	14.48	6.58	–6.88^∗∗∗^	–0.74

**^∗^p < 0.05, ^∗∗^p < 0.01, ^∗∗∗^p < 0.001.**

**TABLE 2:** Chi-squared- and *t*-tests showing the differences between chronic pain patients screening positive and negative for PTSS at a level qualifying for a PTSD diagnosis in the first wave of data collection.

	**Total**		**Screening positive for PTSD**		**Screening negative for PTSD**		
			
	***n***	**%**	***n***	**%**	***n***	**%**	**t/*χ*^2^**
Gender:	101						
Male	36	35.6	5	20.0	31	40.8	
Female	65	64.4	20	80.0	45	59.2	3.54
Age:	101						
18–50	58	57.4	18	72.0	40	52.6	
≥51	43	42.6	7	28.0	36	47.4	2.89
Diagnostic group:	101						
Primary pain	77	76.2	17	68.0	60	78.9	
Secondary pain	24	23.8	8	32.0	16	21.1	1.24
Living situation:	95						
Alone	33	34.7	12	52.2	21	29.2	
With others	62	65.3	11	47.8	51	70.8	4.07^∗^
Marital status:	98						
Unmarried	61	62.2	16	66.7	45	60.8	
Married	37	37.8	8	33.3	29	39.2	0.26
Children:	98						
Have children	71	72.4	19	79.2	52	70.3	
No children	27	27.6	5	20.8	22	29.7	0.72
Work or life situation:	97						
Working/student/military service	39	40.2	7	30.4	32	43.2	
Not working	58	59.8	16	69.6	42	56.8	1.20

	**M**	***SD***	**M**	***SD***	**M**	***SD***	**t/*χ*^2^**

Age (21–83) (*n* = 101)	47.77	15.17	46.16	12.58	48.30	15.97	0.69
NRS pain intensity (0–10) (*n* = 90)	7.29	1.64	8.05	1.36	7.04	1.66	−2.56^∗^
NRS pain distress (0–10) (*n* = 90)	7.88	1.64	8.59	1.22	7.65	1.70	−2.41^∗^
Pain duration in years (0–99) (*n* = 92)	9.96	11.15	9.05	10.90	10.24	11.29	0.44
Self–perceived health (1–4) (*n* = 92)	3.21	0.66	3.41	0.50	3.14	0.69	–1.70
The modified ODI (0–100) (*n* = 101)	42.50	16.74	51.52	16.45	39.53	15.84	–3.25^∗∗^
HSCL–25 (1–4) (*n* = 91)	2.20	0.56	2.55	0.58	2.06	0.51	–3.80^∗∗∗^
PCS (0–52) (*n* = 92)	23.79	11.35	31.09	9.88	21.50	10.85	–3.69^∗∗∗^
GSE (10–40) (*n* = 92)	28.64	6.63	28.00	8.66	28.84	5.91	0.43
IEQ (0–48) (*n* = 92)	22.68	9.85	27.95	8.11	21.03	9.82	–3.00^∗∗^
CFQ (0–11) (*n* = 92)	6.52	3.51	7.77	2.83	6.13	3.62	–1.95
ISI (0–28) (*n* = 92)	15.95	6.92	21.32	5.28	14.26	6.52	–4.62^∗∗∗^

*p < 0.05, **p < 0.01, and ***p < 0.001.

**TABLE 3:** Chi-squared- and *t*-tests showing the differences between chronic pain patients screening positive and negative for PTSS at a level qualifying for a PTSD diagnosis in the second wave of data collection.

	**Total**		**Screening positive for PTSD**		**Screening negative for PTSD**		
			
	***n***	**%**	***n***	**%**	***n***	**%**	***t/χ^2^***
Gender:	475						
Male	177	37.3	35	36.8	142	37.4	
Female	298	62.7	60	63.2	238	62.6	0.01
Age:	488						
18–50	291	59.6	68	70.1	223	57.0	
≥51	197	40.4	29	29.9	168	43.0	5.52^∗^
Diagnostic group:	488						
Primary pain	223	45.7	51	52.6	172	44.0	
Secondary pain	265	54.3	46	47.4	219	56.0	2.31
Living situation:	466						
Alone	135	29.0	26	28.3	109	29.1	
With others	331	71.0	66	71.7	265	70.9	0.03
Marital status:	472						
Unmarried	284	60.2	68	71.6	216	57.3	
Married	188	39.8	27	28.4	161	42.7	6.46^∗^
Children:	473						
Have children	302	63.8	53	55.8	249	65.9	
No children	171	36.2	42	44.2	129	34.1	3.34
Work or life situation:	459						
Working/student/military service	176	38.3	26	28.6	150	40.8	
Not working	283	61.7	65	71.4	218	59.2	4.59^∗^

	**M**	***SD***	**M**	***SD***	**M**	***SD***	***t/χ^2^***

Age (18–92) (*n = * 488)	48.14	15.60	42.63	13.41	48.68	16.0	3.45^∗∗^
NRS pain intensity (0–10) (*n = * 461)	7.15	1.72	7.47	1.58	7.07	1.74	–1.92
NRS pain distress (0–10) (*n = * 463)	7.76	1.69	8.09	1.54	7.68	1.71	−2.04^∗^
Pain duration in years (0–99) (*n = * 465)	7.08	7.60	8.07	7.92	6.85	7.52	–1.36
Self–perceived health (1–4) (*n = * 465)	3.15	0.74	3.49	0.57	3.07	0.75	–4.84^∗∗∗^
The modified ODI (0–100) (*n = * 488)	42.63	17.89	49.66	18.41	40.88	17.34	–4.41^∗∗∗^
HSCL–25 (1–4) (*n = * 464)	2.16	0.59	2.76	0.53	2.02	0.52	–12.01^∗∗∗^
PCS (0–52) (*n = * 463)	24.20	12.56	34.06	11.39	21.95	11.71	–8.70^∗∗∗^
GSE (10–40) (*n = * 461)	28.62	5.54	25.93	4.89	29.24	5.50	5.16^∗∗∗^
IEQ (0–48) (*n = * 461)	24.26	11.42	32.46	9.90	22.35	10.90	–7.92^∗∗∗^
CFQ (0–11) (*n = * 463)	7.10	3.01	8.57	2.30	6.76	3.15	–6.14^∗∗∗^
ISI (0–28) (*n = * 646)	15.32	6.82	18.79	6.72	14.52	6.60	–5.43^∗∗∗^

**^∗^p < 0.05, ^∗∗^p < 0.01, ^∗∗∗^p <0.001.**

### Demographic Factors

Of the total study population, 63% (*n* = 363) were female. The mean age was 47.5 years (*SD*: 15.56). No significant association was found between sex and screening positive for PTSD. The association between participants’ age and screening positive for PTSD did, however, reach statistical significance, both according to age group and actual age. Whether the participants were married or not, and currently working (including military service and being a student) or not, were also significantly associated with PTSS in the study population. On the contrary, the association between screening positive for PTSD and the groups of pain diagnoses (primary pain and secondary pain) did not reach statistical significance, neither did the association between screening positive for PTSD and the categories of secondary pain (chronic cancer-related pain, chronic postsurgical or posttraumatic pain, chronic secondary musculoskeletal pain, chronic secondary visceral pain, chronic neuropathic pain, and chronic secondary headache or orofacial pain). In addition, nor the association between screening positive for PTSD and living status nor being a parent or not reached statistical significance. For further details, see Table 1. For details about the first and the second wave of data collection, see Tables 2 and 3.

### Pain Intensity, Pain Bothersomeness, and Disability Level

Screening positive for PTSD was significantly associated with having higher pain intensity and more pain bothersomeness. There was no significant difference in pain duration between the patients who screened positive for PTSD compared to patients in who screened negative for PTSD. There were, however, differences in how the participants perceived their overall health condition, with those who screened positive for PTSD perceiving their health conditions as poorer than those who screened negative. In addition, screening positive for PTSD was significantly associated with higher disability scores. Further details are presented in Table 1 (total study population), Table 2 (wave one), and Table 3 (wave two).

### Psychological Distress, Pain Catastrophizing, Perceived Self-Efficacy, and Injustice Experiences

PTSS at a level qualifying for a PTSD diagnosis were significantly associated with higher levels of psychological distress. Screening positive for PTSD was also significantly associated with more pain catastrophizing, as well as with the participants’ perceived level of self-efficacy. Further, the patients’ experiences of injustice were significantly associated with high levels of PTSS. For further details, see Table 1. For details regarding wave one and wave two of data collection, see Tables 2 and 3.

### Fatigue and Sleep Difficulties

Screening positive for PTSD was significantly associated with fatigue in the study sample. Significant group differences between patients with chronic pain and high levels of PTSS and patients with chronic pain alone were also found on sleep disturbances. See Table 1 for additional information about the total study sample, and Tables 2 and 3 for information about wave one and wave two separately.

### Confounding Variables

The associations between screening positive for PTSD and adverse outcomes were controlled for confounding effects from age, marital status and work or life situation. No confounding effects were found. Details are presented in Table 4.

**TABLE 4:** Univariate and multivariate associations (odds ratios) between screening positive for PTSD and adverse outcomes.

	**Univariate associations**		**Multivariate model^*a*^**	
	**OR (95% CI)**	***p*-value**	**OR (95% CI)**	***p*-value**
NRS Pain intensity (0–10)	1.20(1.06-1.37)	<0.01	1.26(1.09-1.46)	<0.01
NRS Pain bothersomeness (0–10)	1.22(1.06-1.40)	<0.01	1.18(1.02-1.37)	<0.05
Self–Perceived Health (1–4)	2.33(1.66-3.27)	<0.001	2.35(1.63-3.41)	<0.001
The Modified ODI (0–100)	1.03(1.02-1.04)	<0.001	1.04(1.02-1.05)	<0.001
HSCL–25 (1–4)	9.72(6.09-15.51)	<0.001	8.92(5.43-14.64)	<0.001
PCS (0–52)	1.09(1.07-1.11)	<0.001	1.10(1.07-1.12)	<0.001
GSE (10–40)	0.92(0.89-0.95)	<0.001	0.92(0.86-0.96)	<0.001
IEQ (0–48)	1.09(1.06-1.11)	<0.001	1.08(1.06-1.11)	<0.001
CFQ (0–11)	1.24(1.14-1.35)	<0.001	1.24(1.13-1.36)	<0.001
ISI (0–28)	1.13(1.09-1.17)	<0.001	1.14(1.09-1.19)	<0.001

*^*a*^Adjusted for age, marital status and work or life situation.*

## Discussion

The main objective of this study was to examine the prevalence of PTSS in patients with chronic pain conditions consecutively accepted to pain assessment at a Norwegian university hospital outpatient pain clinic. The study demonstrated a high rate of exposure to potentially traumatic events in this population. This finding may not be too surprising, as for some patients, their pain may have been caused by a traumatic event. The study further showed that one-fifth of patients assessed at the clinic reported PTSS at a level consistent with a PTSD diagnosis. Earlier studies have shown widely varying prevalence rates of PTSD among patients with chronic pain conditions (Hickling and Blanchard, 1992; Siqveland et al., 2017). This could be due to different patient populations, the use of different measurements methods or different diagnostic criteria, or a combination of these. This study does, in line with two other Scandinavian studies (Andersen et al., 2012; Akerblom et al., 2017), present a high prevalence rate of severe PTSS in patients with chronic pain. Despite a slightly lower rate, the data clearly demonstrates the extent to which chronic pain and PTSS co-occur, and the importance of PTSS as a significant issue among patients in outpatient pain clinics is highlighted.

The second objective of this study was to examine whether screening positive for PTSD is associated with adverse outcomes, such as pain severity, disability and distress. The results showed a significantly higher symptom burden among chronic pain patients with high levels of PTSS compared to chronic pain patients with low levels of PTSS. PTSS were associated with more severe pain and higher levels of disability, psychological distress, pain catastrophizing, and perceived injustice, as well as higher levels of fatigue and sleep difficulties. In addition, screening positive for PTSD was associated with lower levels of self-efficacy.

Patients with chronic pain and comorbid high levels of PTSS reported more severe pain, which is in line with previous studies (Sherman et al., 2000). The effect size is, however, small to medium for both pain intensity and pain bothersomeness. Whether this difference in pain intensity is clinically significant could be argued, as there is a large variability in minimum clinically important differences (MCID) in chronic pain (Frahm Olsen et al., 2018). Also, measures of MCID for chronic pain are context-dependent and sensitive to clinical and methodological characteristics (Beaton et al., 2002).

The presence of severe PTSS did also affect how the participants perceived their overall health, and the strength of the association between PTSS and perception of health was found to be medium to large. As a larger portion of participants who screened positive for PTSD reported their health to be “not good” or “poor” compared to those who screened negative for PTSD, it may suggest that PTSS add to the total symptom burden for patients with chronic pain conditions. Although there may be some symptom overlap between the two conditions, the majority of PTSS will represent more symptoms, which in turn may add to the perception of impairment. It should be noted that the scale used to assess self-perceived health has some limitations. A Likert scale will have low discriminating power, and it will not be able to provide any detailed insights.

Screening positive for PTSD was associated with higher levels of pain-related disability, and the association was of medium to large strength. This is consistent with previous research suggesting that PTSD is disabling by definition, through the avoidance of stimuli associated with the traumatic event (Geisser et al., 1996). Further, elevated levels of affective distress associated with severe PTSS may constitute an indirect relationship between PTSD and heightened disability levels. Pain may act as a reminder of the traumatic event, eliciting an arousal response as well as other symptoms of PTSD (Sharp and Harvey, 2001). This arousal may then trigger avoidance of activities that cause pain, and of trauma memories accompanied by the arousal. When sensations associated with the trauma are avoided, levels of distress and disability may be exacerbated. Also, disability may, like chronic pain, act as a reminder of the trauma and contribute to the negative development. This mutual maintenance is described in one of the most influential models of the relationship between chronic pain and PTSD; the Mutual Maintenance Model (Sharp and Harvey, 2001). The model states that chronic pain and PTSD are maintained by seven specific factors: (1) Attentional and reasoning biases, (2) anxiety sensitivity, (3) reminders of the trauma, (4) avoidance, (5) depression and reduced activity levels, (6) anxiety and pain perception, and (7) cognitive demand from symptoms limiting use of adaptive strategies.

Avoidance behavior is found to decrease pain-related self-efficacy (Waddell et al., 1993). Self-efficacy is belief in one’s own abilities to perform a task or achieve a goal (Bandura, 1977), and it is largely determined by earlier experiences. Self-efficacy may strongly influence pain tolerance (Keefe et al., 1997) and overall functioning (Woby et al., 2005), perhaps through motivation for treatment and health-promoting behavior (Gatchel et al., 2007). Compared to fear-avoidance behavior, self-efficacy accounts for a much larger portion of the variance in disability scores in patients with chronic pain conditions (Ayre and Tyson, 2001). A significant association between high levels of PTSS and level of perceived self-efficacy was found in the current study, and it had medium strength. This might suggest that patients who struggle with both chronic pain and PTSS are so affected and possibly overwhelmed by the extra burden PTSS constitute in their daily life, that their belief in own abilities to overcome the challenges are weakened.

Patients with severe PTSS reported a significantly higher level of affective distress compared to patients with chronic pain alone. Psychological distress is experienced by a large portion of patients with chronic pain conditions in general, but the prevalence of such symptoms is even higher when severe PTSS are present. The effect size was found to be very large, suggesting that this association should be addressed in clinical settings. Anxiety is one of the factors proposed to be mutually maintaining for comorbid chronic pain conditions and PTSD (Sharp and Harvey, 2001), and the subjective experience of pain is found to be aggravated by high levels of anxiety (Difede et al., 1997). As anxiety is central in PTSD (American Psychiatric Association, 2013), this may suggest that PTSD increases pain intensity and pain bothersomeness by the associated anxiety. More pain may then increase passivity, which in turn may cause higher disability and higher levels of affective distress (Waddell et al., 1993).

Depression is one of the suggested mutually maintaining factors that has gained most attention. In patients with chronic pain conditions, depression has been linked to more severe pain, increased pain behavior and higher interference in daily living (Haythornthwaite et al., 1991), as well as poorer treatment outcomes (Blanchard et al., 1982). A high comorbidity rate has been found between depression and both chronic pain and PTSD (Roth et al., 2008). The association between PTSS and psychological distress found in this study may, however, to some degree be due to similarities between the diagnostic criteria for PTSD and Major Depressive Disorder (American Psychiatric Association, 2013), or depressive symptoms may be developed as a consequence of struggling with PTSS. Also, aspects of depression (for example, lethargy and fatigue) and the following reduction in activity may maintain both conditions (Sharp and Harvey, 2001).

More recently it has been suggested that the relationship between chronic pain and comorbid PTSD and depression may be modulated by the individual’s perception of injustice (Scott and Sullivan, 2012). Compared to patients with chronic pain alone, patients with both chronic pain and severe PTSS reported a significantly higher level of perceived injustice in this study, and the results indicate a strong association between the two. This is one of very few studies to investigate the prevalence of significant clinical levels of perceived injustice in patients consecutively accepted to pain assessment at a hospital outpatient pain clinic. However, studies of other pain populations have suggested that high levels of perceived injustice are associated with a lower probability of return to work after injury (Sullivan et al., 2008) and poorer pain-related outcomes (Scott et al., 2014). As experiences of injustice have been associated with both pain catastrophizing and self-reported disability, these factors may share mechanisms that affect the persistence of PTSS (Sullivan et al., 2009). Further, perceived injustice may not decrease after standard multidisciplinary treatment programs, suggesting a need for treatments targeting this experience specifically (Sullivan et al., 2009).

A strong association between high levels of PTSS and pain catastrophizing was also found in the current study, as patients with chronic pain and PTSS reported significantly higher levels of pain catastrophizing than patients with chronic pain alone. This finding is in line with previous studies (Sullivan et al., 2009), and also with the Mutual Maintenance Model (Sharp and Harvey, 2001) that proposes that anxiety sensitivity acts as a mutually maintaining factor of comorbid chronic pain and PTSD. Anxiety sensitivity is seen as a vulnerability factor for misinterpreting physical symptoms associated with pain and PTSD as overly threatening (catastrophizing), and it is thought to demand a high degree of cognitive capacity (Crombez et al., 1998). PTSS (for example, intrusive memories) may, in line with pain catastrophizing demand much cognitive capacity. As cognitive strategies requiring attentional control may reduce pain perception, the cognitive capacity left to use adaptive coping strategies may be very limited. As a result of this, the pain may be experienced as more severe (Bryant et al., 1999). Pain catastrophizing is found to influence the degree to which patients with chronic pain adjust to their pain and it is associated with negative outcomes for this patient group (Vlaeyen and Linton, 2000).

Patients screening positive for PTSD further reported a higher level of fatigue compared to patients screening negative for PTSD, and the effect size was medium. Patients with chronic pain conditions frequently report symptoms of fatigue (Salvetti Mde et al., 2013), and fatigue is found to be independently associated with depression and self-efficacy in this patient group (Salvetti Mde et al., 2013). However, when severe PTSS are present, the level of fatigue increases significantly. One explanation for this may be that PTSS cause, or exacerbate, fatigue by adding an extra burden to the patients’ symptom load. Hence, it may be more difficult to perform health-promoting activities and adhere to treatments. Another explanation may be that both chronic pain conditions and fatigue may develop after encountering a stressful event (Kang et al., 2003). Also, biological predispositions have been suggested as a risk factor for developing both disorders after exposure to trauma (van Zuiden et al., 2012).

Sleep difficulties were also seen more often among patients with high levels of PTSS compared to patients with low levels of such symptoms. The association between sleep problems and severe PTSS were strong, suggesting it should not be overlooked in clinical settings. Difficulties with falling or staying asleep are known to be frequently reported by patients with chronic pain, and it is often considered to be one of the most distressing challenges in the patients’ life (Tang, 2008). Patients with chronic pain and sleep disturbances report more severe pain and psychological symptoms, such as anxiety and depression (Tang, 2008). PTSD is also known to cause sleep disturbances, as a large majority (70–91%) of patients with PTSD report some kind of sleeping difficulties (Maher et al., 2006). These difficulties affect both the development and severity of PTSS, as well as the overall functioning and quality of life (Maher et al., 2006). Sleep disturbances may then have a negative effect on both chronic pain and PTSD, and sleep difficulties caused by one of the disorders may aggravate the other condition. The relationship between chronic pain, PTSD and sleep difficulties may be tri-directional, and not fully understood.

The third objective of this study was to examine whether PTSS prevalence differ between patients with a primary versus a secondary pain condition. The results showed that there were no statistically significant differences between patients screening positive for PTSD and patients screening negative for PTSD when it comes to group of pain conditions (primary versus secondary) nor to the categories of secondary pain conditions (chronic cancer-related pain, chronic postsurgical or posttraumatic pain, chronic secondary musculoskeletal pain, chronic secondary visceral pain, chronic neuropathic pain, and chronic secondary headache or orofacial pain). Hence, the data suggest that the etiology of PTSS and pain diagnosis are unrelated, at least at group level. The course and outcome of the two conditions might still be affected by the presence of the other [as outlined in the Mutual Maintenance Model (Sharp and Harvey, 2001)], but it appears that the prevalence of PTSD is no higher in patients with chronic postsurgical or posttraumatic pain compared to patients with other categories of secondary chronic pain.

These findings might be surprising, as earlier studies have reported that pain diagnoses and characteristics are indeed related to symptoms of PTSD (de Leeuw et al., 2005). The lack of differences in our study may be due to the broad pain classification we applied, which might not have been sensitive enough to identify potential differences in specific pain diagnoses. The lack of differences found may also be due to the very limited number of participants in some of the pain categories. The number of patients with chronic postsurgical or posttraumatic pain is low in this study, and it is also not possible to distinguish between patients suffering from postsurgical versus posttraumatic pain. As the literature suggests a higher comorbidity between pain and PTSD when both conditions are caused by a shared traumatic event (Koren et al., 2005), this is an important point to consider. Further, the complexity of chronic pain may also explain these findings. As the study population is a very heterogenous group, many patients have suffered from chronic pain for several years, and many patients have more than one diagnosis, the differences in PTSS prevalence may not become evident.

The study has some limitations. In line with similar studies (for example, Andersen et al., 2012; Akerblom et al., 2017), we report on PTSS assessed by self-report questionnaires. A PTSD diagnosis can never be set based on such questionnaires alone, and thereby the results will only be able to provide insight into the patient’s experience of the presence or absence of each symptom listed on IES-R or PCL-5. Hence, the authors were not able to ensure that the individuals that screened positive for PTSD fulfilled the diagnostic criteria for a PTSD diagnosis. There are also differences in the diagnostic criteria for PTSD between the fourth and fifth version of DSM (Pai et al., 2017). In this study, we used screening questionnaires for PTSD based on both versions, which could raise questions about the accuracy of the PTSD prevalence rate found. Studies do, however, suggest that IES-R and PCL-5 have a strong positive correlation (Ashbaugh et al., 2016). A further limitation is that a large portion of the participants (35.3%) reported being exposed to a traumatic event listed as “Other” on LEC or SLESQ, and “Other” was regarded as fulfillment of the A Criterion in DSM-IV (American Psychiatric Association, 1994) and DSM-5 (American Psychiatric Association, 2013). As a result, the authors cannot exclude the possibility that the event(s) the participants had in mind did not qualify as the A Criterion. Also, when completing IES-R and PCL-5, the patients were instructed to have the event causing most distress in the last weeks in mind. The patients’ PTSS may, however, not have been caused by one single event. Moreover, the study design is cross-sectional, which does not allow for conclusions regarding causality. While causal direction does not affect the patients’ experience of the symptoms, it is of great interest to clinicians and researchers.

As the main objective of this study was to examine the prevalence of PTSS in a chronic pain population, the challenges of using cut-off scores for categorizing patients into the “with PTSD” and the “without PTSD” group should also be emphasized. While the cut-off scores used in this study have been well-validated for use in other populations, it is still uncertain which cut-off score should be applied when screening for PTSS in pain populations. To our knowledge, only one previous study has investigated the optimal cut-off score on PCL when used in this patient group, and they found it to be 44 (Dunn et al., 2011). The cut-off score of 33 was found to have a specificity of .60 in the mentioned study, which is lower than the specificity found in studies validating cut-off scores from 31 to 33 in other populations (Blevins et al., 2015; Bovin et al., 2016). Also, studies suggest that the prevalence of PTSD may be overestimated in pain populations unless overlapping symptoms between chronic pain and PTSD are considered (Fishbain et al., 2017). As we chose to proceed with a cut-off score of 33 there may be an increased risk of false positives in the study, and hence the reported prevalence rate of patients screening positive for PTSD may be too high.

Questions have been raised regarding the strength of standardized screening instruments when used to assess potentially traumatic life events and PTSS among patients with chronic pain conditions. An interesting direction for further research would be to explore the appropriateness of such questionnaires when used in this patient population, for example by asking patients to complete both screening questionnaires and then clinical interviews, to confirm or discard the results from the screening test. Only by doing this will it become clear whether screening questionnaires for PTSD perform as well in this population as in other clinical populations. It would also be interesting to explore the directionality of the observed comorbidity between chronic pain conditions and PTSS. Longitudinal studies could provide important insights into whether chronic pain may be a vulnerability factor for developing PTSS, or if PTSS may play a role in developing a chronic pain condition. Future research should also aim to increase knowledge of how physiological and psychological factors affect both chronic pain and PTSS, and the interaction between them, as this would lay the foundation for developing better interventions.

The finding that one-fifth of patients consecutively accepted for pain assessment at a university hospital outpatient pain clinic reported a significant level of PTSS emphasizes the need for increased attention on PTSS in this patient group. Further, the results demonstrate associations between PTSS and a range of psychological factors in patients with chronic pain. Patients with both conditions reported a significantly higher symptom load, and the potential impact on the life of these patients is major. Whether the patient suffered from a primary pain condition or a condition that is related to chronic pain was, however, not associated with a high level of PTSS. Even though the design of this study does not allow us to draw any conclusions regarding causal direction, the significant differences found strongly suggest that extra attention should be paid to patients with chronic pain conditions presenting with PTSS.

## Data Availability Statement

The datasets generated for this study are available on request to the corresponding author.

## Ethics Statement

The studies involving human participants were reviewed and approved by The Data Protection Officer at Oslo University Hospital (ref. 2014/1309). The patients/participants provided their written informed consent to participate in this study.

## Author Contributions

SR was the principal investigator of the study, while LL was the coordinator. LL and SR drafted the manuscript, while L-PG revised the manuscript for critical intellectual content. All authors contributed to the development of the study design, read, and approved the final manuscript.

## Conflict of Interest

The authors declare that the research was conducted in the absence of any commercial or financial relationships that could be construed as a potential conflict of interest.

## References

[B1] Akerblom S. Perrin S. Rivano Fischer M. McCracken L. M. (2017). The impact of PTSD on functioning in patients seeking treatment for chronic pain and validation of the posttraumatic diagnostic scale. *Int. J. Behav. Med.* 24 249–259. 10.1007/s12529-017-9641-8 28194719 PMC5344943

[B2] American Psychiatric Association (1994). *Diagnostic and Statistical Manual of Mental Disorders*, 4th Edn Washington, DC: American Psychiatric Association.

[B3] American Psychiatric Association (2013). *Diagnostic and Statistical Manual of Mental Disorders*, 5th Edn Washington, DC: American Psychiatric Association.

[B4] Andersen T. Andersen P. Vakkala M. Elklit A. (2012). The traumatised chronic pain patient-prevalence of posttraumatic stress disorder- PTSD and pain sensitization in two scandinavian samples referred for pain rehabilitation. *Scand. J. Pain* 3 39–43. 10.1016/j.sjpain.2011.10.001 29913766

[B5] Ashbaugh A. R. Houle-Johnson S. Herbert C. El-Hage W. Brunet A. (2016). Psychometric validation of the english and french versions of the posttraumatic stress disorder checklist for DSM-5 (PCL-5). *PLoS One* 11 e0161645 10.1371/journal.pone.0161645 PMC5056703 27723815

[B6] Asmundson G. J. Coons M. J. Taylor S. Katz J. (2002). PTSD and the experience of pain: research and clinical implications of shared vulnerability and mutual maintenance models. *Can. J. Psychiatry* 47 930–937. 10.1177/070674370204701004 12553128

[B7] Asmundson G. J. Jacobson S. J. Allerdings M. D. Norton G. R. (1996). Social phobia in disabled workers with chronic musculoskeletal pain. *Behav. Res. Ther.* 34 939–943. 10.1016/s0005-7967(96)00055-1 8990546

[B8] Ayre M. Tyson G. A. (2001). The role of self-efficacy and fear-avoidance beliefs in the prediction of disability. *Austr. Psychol.* 36 250–253. 10.1080/00050060108259663

[B9] Baker D. J. Pynsent P. B. Fairbank J. C. T. (1990). “The Oswestry disability index revisited: its reliability, repeatability and validity, and a comparison with the St. Thomas’ disability index,” in *Back Pain: New Approaches to Rehabilitation and Education*, eds Roland M. O. Jenner R. (Manchester: Manchester University Press), 175–181.

[B10] Bandura A. (1977). Self-efficacy: toward a unifying theory of behavioral change. *Psychol. Rev.* 84 191–215. 10.1037/0033-295x.84.2.191 847061

[B11] Banks S. M. Kerns R. D. (1996). Explaining the high rates of depression in chronic pain: a stress-diathesis framework. *Psychol. Bull.* 119 95–110. 10.1037/0033-2909.119.1.95

[B12] Bastien C. H. Vallieres A. Morin C. M. (2001). Validation of the Insomnia Severity Index as an outcome measure for insomnia research. *Sleep Med.* 2 297–307. 10.1016/s1389-9457(00)00065-4 11438246

[B13] Beaton D. E. Boers M. Wells G. A. (2002). Many faces of the minimal clinically important difference (MCID): a literature review and directions for future research. *Curr. Opin. Rheumatol* 14 109–114. 10.1097/00002281-200203000-00006 11845014

[B14] Beckham J. C. Crawford A. L. Feldman M. E. Kirby A. C. Hertzberg M. A. Davidson J. R. (1997). Chronic posttraumatic stress disorder and chronic pain in Vietnam combat veterans. *J. Psychos. Res.* 43 379–389. 10.1016/s0022-3999(97)00129-3 9330237

[B15] Benyamini Y. (2011). Why does self-rated health predict mortality? An update on current knowledge and a research agenda for psychologists. *Psychol. Health* 26 1407–1413. 10.1080/08870446.2011.621703 22111660

[B16] Bjornvatn B. (2016). *Chalder Fatigue Questionnaire Norwegian version.* Available online at: http://www.helse-bergen.no/no/OmOss/Avdelinger/sovno/Documents/Fatigue_Questionnaire_norsk%202.pdf (accessed August 26, 2018).

[B17] Blake D. D. Weathers F. W. Nagy L. M. Kaloupek D. G. Gusman F. D. Charney D. S. (1995). The development of a clinician-administered PTSD Scale. *J. Trauma Stress* 8 75–90. 7712061 10.1007/BF02105408

[B18] Blanchard E. B. Andrasik F. Neff D. F. Arena J. G. Ahles T. A. Jurish S. E. (1982). Biofeedback and relaxation training with three kinds of headache: treatment effects and their prediction. *J. Consult. Clin. Psychol.* 50 562–575. 10.1037/0022-006x.50.4.562 6749918

[B19] Blevins C. A. Weathers F. W. Davis M. T. Witte T. K. Domino J. L. (2015). The posttraumatic stress disorder checklist for DSM-5 (PCL-5): development and initial psychometric evaluation. *J. Trauma Stress* 28 489–498. 10.1002/jts.22059 26606250

[B20] Bovin M. J. Marx B. P. Weathers F. W. Gallagher M. W. Rodriguez P. Schnurr P. P. (2016). Psychometric properties of the PTSD checklist for diagnostic and statistical manual of mental disorders-fifth edition (PCL-5) in veterans. *Psychol. Assess.* 28 1379–1391. 10.1037/pas0000254 26653052

[B21] Bryant R. A. Marosszeky J. E. Crooks J. Baguley I. J. Gurka J. A. (1999). Interaction of posttraumatic stress disorder and chronic pain following traumatic brain injury. *J. Head Trauma Rehabil.* 14 588–594. 10671704 10.1097/00001199-199912000-00007

[B22] Chalder T. Berelowitz G. Pawlikowska T. Watts L. Wessely S. Wright D. (1993). Development of a fatigue scale. *J. Psychosom. Res.* 37 147–153. 8463991 10.1016/0022-3999(93)90081-p

[B23] Creamer M. Bell R. Failla S. (2003). Psychometric properties of the impact of event scale - revised. *Behav. Res. Ther.* 41 1489–1496. 10.1016/j.brat.2003.07.010 14705607

[B24] Creavin S. T. Dunn K. M. Mallen C. D. Nijrolder I. van der Windt D. A. (2010). Co-occurrence and associations of pain and fatigue in a community sample of dutch adults. *Eur. J. Pain* 14 327–334. 10.1016/j.ejpain.2009.05.010 19540139

[B25] Crombez G. Eccleston C. Baeyens F. Eelen P. (1998). When somatic information threatens, catastrophic thinking enhances attentional interference. *Pain* 75 187–198. 10.1016/s0304-3959(97)00219-4 9583754

[B26] de Leeuw R. Schmidt J. E. Carlson C. R. (2005). Traumatic stressors and post-traumatic stress disorder symptoms in headache patients. *Headache* 45 1365–1374. 10.1111/j.1526-4610.2005.00269.x 16324169

[B27] Derogatis L. R. Lipman R. S. Rickels K. Uhlenhuth E. H. Covi L. (1974). The hopkins symptom checklist (HSCL): a self-report symptom inventory. *Behav. Sci.* 19 1–15. 10.1002/bs.3830190102 4808738

[B28] Difede J. Jaffe A. B. Musragi G. Perry S. Yurt R. (1997). Determinants of pain expression in hospitalized burn patients. *Pain* 72 245–251. 10.1016/s0304-3959(97)00045-6 9272809

[B29] Dunn A. S. Julian T. Formolo L. R. Green D. R. (2011). Preliminary analysis of posttraumatic stress disorder screening within specialty clinic setting for OIF/OEF veterans seeking care for neck or back pain. *J. Rehabil. Res. Dev.* 48 493–502. 21674400 10.1682/jrrd.2010.05.0104

[B30] Eid J. Larsson G. Johnsen B. H. Laberg J. C. Bartone P. T. Carlstedt B. (2009). Psychometric properties of the Norwegian Impact of Event Scale-revised in a non-clinical sample. *Nord. J. Psychiatry* 63 426–432. 10.1080/08039480903118190 19688636

[B31] Elhai J. D. Gray M. J. Kashdan T. B. Franklin C. L. (2005). Which instruments are most commonly used to assess traumatic event exposure and posttraumatic effects?: a survey of traumatic stress professionals. *J. Trauma Stress* 18 541–545. 10.1002/jts.20062 16281252

[B32] Fernandes L. Storheim K. Lochting I. Grotle M. (2012). Cross-cultural adaption and validation of the Norwegian pan catastrophizing scale in patients with low back pain. *BMC Musculoskelet Disord.* 13:111 10.1186/1471-2474-13-111 PMC3407790 22726668

[B33] Fishbain D. A. Pulikal A. Lewis J. E. Gao J. (2017). Chronic pain types differ in their reported prevalence of post -traumatic stress disorder (PTSD) and there is consistent evidence that chronic pain is associated with PTSD: an evidence-based structured systematic review. *Pain Med.* 18 711–735. 27188666 10.1093/pm/pnw065

[B34] Fong T. C. Chan J. S. Chan C. L. Ho R. T. Ziea E. T. Wong V. C. (2015). Psychometric properties of the Chalder Fatigue Scale revisited: an exploratory structural equation modeling approach. *Qual. Life Res.* 24 2273–2278. 10.1007/s11136-015-0944-4 25688039 PMC4529874

[B35] Frahm Olsen M. Bjerre E. Hansen M. D. Tendal B. Hilden J. Hrobjartsson A. (2018). Minimum clinically important differences in chronic pain vary considerably by baseline pain and methodological factors: systematic review of empirical studies. *J. Clin. Epidemiol.* 101 87.e2–106.e2. 29793007 10.1016/j.jclinepi.2018.05.007

[B36] Gatchel R. J. Peng Y. B. Peters M. L. Fuchs P. N. Turk D. C. (2007). The biopsychosocial approach to chronic pain: scientific advances and future directions. *Psychol. Bull.* 133 581–624. 10.1037/0033-2909.133.4.581 17592957

[B37] Geisser M. E. Roth R. S. Bachman J. E. Eckert T. A. (1996). The relationship between symptoms of post-traumatic stress disorder and pain, affective disturbance and disability among patients with accident and non-accident related pain. *Pain* 66 207–214. 10.1016/0304-3959(96)03038-2 8880842

[B38] Goodman L. A. Corcoran C. Turner K. Yuan N. Green B. L. (1998). Assessing traumatic event exposure: general issues and preliminary findings for the stressful life events screening questionnaire. *J. Trauma Stress* 11 521–542. 10.1023/a:1024456713321 9690191

[B39] Gray M. J. Litz B. T. Hsu J. L. Lombardo T. W. (2004). Psychometric properties of the life events checklist. *Assessment* 11 330–341. 10.1177/1073191104269954 15486169

[B40] Grotle M. Brox J. I. Vollestad N. K. (2003). Cross-cultural adaptation of the norwegian versions of the roland-morris disability questionnaire and the oswestry disability index. *J. Rehabil. Med.* 35 241–247. 10.1080/16501970306094 14582557

[B41] Halvorsen J. O. Stenmark H. (2010). Narrative exposure therapy for posttraumatic stress disorder in tortured refugees: a preliminary uncontrolled trial. *Scand. J. Psychol.* 51 495–502. 10.1111/j.1467-9450.2010.00821.x 20487410

[B42] Haythornthwaite J. A. Sieber W. J. Kerns R. D. (1991). Depression and the chronic pain experience. *Pain* 46 177–184. 10.1016/0304-3959(91)90073-7 1749640

[B43] Heir T. (2013). *Norwegian Translation of the PTSD Checlist for DSM-5 (PCL-5).* Oslo: The Norwegian Centre for Violence and Traumatic Stress Studies.

[B44] Hickling E. J. Blanchard E. B. (1992). Post-traumatic stress disorder and motor vehicle accidents. *J. Anxiety Disord.* 6 285–291. 10.1016/0887-6185(92)90040-e

[B45] Idler E. L. Benyamini Y. (1997). Self-rated health and mortality: a review of twenty-seven community studies. *J. Health Soc. Behav.* 38 21–37. 9097506

[B46] Jylha M. (2009). What is self-rated health and why does it predict mortality? Towards a unified conceptual model. *Soc. Sci. Med.* 69 307–316. 10.1016/j.socscimed.2009.05.013 19520474

[B47] Kang H. K. Natelson B. H. Mahan C. M. Lee K. Y. Murphy F. M. (2003). Post-traumatic stress disorder and chronic fatigue syndrome-like illness among Gulf War veterans: a population-based survey of 30,000 veterans. *Am. J. Epidemiol.* 157 141–148. 10.1093/aje/kwf187 12522021

[B48] Keefe F. J. Lefebvre J. C. Maixner W. Salley A. N. Jr. Caldwell D. S. (1997). Self-efficacy for arthritis pain: relationship to perception of thermal laboratory pain stimuli. *Arthritis Care Res.* 10 177–184. 10.1002/art.1790100305 9335629

[B49] Koren D. Norman D. Cohen A. Berman J. Klein E. M. Increased P. T. S. D. (2005). risk with combat-related injury: a matched comparison study of injured and uninjured soldiers experiencing the same combat events. *Am. J. Psychiatry* 162 276–282. 15677591 10.1176/appi.ajp.162.2.276

[B50] Ljoså T. M. Jacobsen H. B. Granan L. P. Reme S. E. (2015). *Rapport fra Oversettelsen av den Norske Versjonen av Injustice Expereince Questionnaire - IEQ, R.C.f. Pain.* Oslo: Oslo University Hospital.

[B51] Loge J. H. Ekeberg O. Kaasa S. (1998). Fatigue in the general Norwegian population: normative data and associations. *J. Psychosom. Res.* 45 53–65. 9720855 10.1016/s0022-3999(97)00291-2

[B52] Maher M. J. Rego S. A. Asnis G. M. (2006). Sleep disturbances in patients with post-traumatic stress disorder: epidemiology, impact and approaches to management. *CNS Drugs* 20 567–590. 10.2165/00023210-200620070-00003 16800716

[B53] Morin C. M. Belleville G. Belanger L. Ivers H. (2011). The Insomnia Severity Index: psychometric indicators to detect insomnia cases and evaluate treatment response. *Sleep* 34 601–608. 10.1093/sleep/34.5.601 21532953 PMC3079939

[B54] Nilsson M. H. Hagell P. Iwarsson S. (2015). Psychometric properties of the General Self-Efficacy Scale in Parkinson’s disease. *Acta Neurol. Scand.* 132 89–96. 10.1111/ane.12368 25630440

[B55] Otis J. D. Keane T. M. Kerns R. D. (2003). An examination of the relationship between chronic pain and post-traumatic stress disorder. *J. Rehabil. Res. Dev.* 40 397–405. 15080224 10.1682/jrrd.2003.09.0397

[B56] Pai A. Suris A. M. North C. S. (2017). Posttraumatic stress disorder in the DSM-5: controversy, change, and conceptual considerations. *Behav. Sci.* 7 7 10.3390/bs7010007 PMC5371751 28208816

[B57] Palyo S. A. Beck J. G. (2005). Post-traumatic stress disorder symptoms, pain, and perceived life control: associations with psychosocial and physical functioning. *Pain* 117 121–127. 10.1016/j.pain.2005.05.028 16099099 PMC1363367

[B58] Roth R. S. Geisser M. E. Bates R. (2008). The relation of post-traumatic stress symptoms to depression and pain in patients with accident-related chronic pain. *J. Pain* 9 588–596. 10.1016/j.jpain.2008.01.333 18343728

[B59] Røysamb E. Schwarzer R. Jerusalem M. (1998). *Norwegian Version of the General Perceived Self-Efficacy Scale.* Available online at: http://userpage.fu-berlin.de/~health/norway.htm. (accessed August 26, 2018).

[B60] Russek L. Gardner S. Maguire K. Stevens C. Brown E. Z. Jayawardana V. (2015). A cross-sectional survey assessing sources of movement-related fear among people with fibromyalgia syndrome. *Clin. Rheumatol.* 34 1109–1119. 10.1007/s10067-014-2494-5 24481649

[B61] Salvetti Mde G. Pimenta C. A. Braga P. E. McGillion M. (2013). Prevalence of fatigue and associated factors in chronic low back pain patients. *Rev. Lat. Am. Enfermagem* 21 12–19. 23459886 10.1590/s0104-11692013000700003

[B62] Sandanger I. Moum T. Ingebrigtsen G. Dalgard O. S. Sorensen T. Bruusgaard D. (1998). Concordance between symptom screening and diagnostic procedure: the hopkins symptom checklist-25 and the composite international diagnostic interview I. *Soc. Psychiatry Psychiatr. Epidemiol.* 33 345–354. 10.1007/s001270050064 9689897

[B63] Schwarzer R. Jerusalem M. (1995). “Generalized self-efficacy scale, in measures in health psychology: a user’s portfolio,” in *Causal and Control Beliefs*, eds Weinman J. Wright S. F. Johnston M. (Windsor: NFER-NELSON), 35–37.

[B64] Scott W. McCracken L. M. Trost Z. (2014). A psychological flexibility conceptualisation of the experience of injustice among individuals with chronic pain. *Br. J. Pain* 8 62–71. 10.1177/2049463713514736 26516537 PMC4590133

[B65] Scott W. Sullivan M. (2012). Perceived injustice moderates the relationship between pain and depressive symptoms among individuals with persistent musculoskeletal pain. *Pain Res. Manag.* 17 335–340. 10.1155/2012/501260 23061084 PMC3465094

[B66] Sharp T. J. Harvey A. (2001). Chronic pain and post-traumatic stress disorder: mutual maintenance? *Clin. Psychol. Rev.* 21 857–877. 10.1016/s0272-7358(00)00071-4 11497210

[B67] Sherman J. J. Turk D. C. Okifuji A. (2000). Prevalence and impact of posttraumatic stress disorder-like symptoms on patients with fibromyalgia syndrome. *Clin. J. Pain* 16 127–134. 10.1097/00002508-200006000-00006 10870725

[B68] Siqveland J. Hussain A. Lindstrom J. C. Ruud T. Hauff E. (2017). Prevalence of posttraumatic stress disorder in persons with chronic pain: a meta-analysis. *Front. Psychiatry* 8 164. 10.3389/fpsyt.2017.00164 PMC5603802 28959216

[B69] Smith M. T. Haythornthwaite J. A. (2004). How do sleep disturbance and chronic pain inter-relate? Insights from the longitudinal and cognitive-behavioral clinical trials literature. *Sleep Med. Rev.* 8 119–132. 10.1016/s1087-0792(03)00044-3 15033151

[B70] Strand B. H. Dalgard O. S. Tambs K. Rognerud M. (2003). Measuring the mental health status of the Norwegian population: a comparison of the instruments SCL-25, SCL-10, SCL-5 and MHI-5 (SF-36). *Nord. J. Psychiatry* 57 113–118. 10.1080/08039480310000932 12745773

[B71] Sullivan M. J. (2008a). *The Pain Catastrophizing Scale User Manual.* Available online at: http://sullivan-painresearch.mcgill.ca/pdf/ieq/IEQManual.pdf (accessed August 26, 2018).

[B72] Sullivan M. J. (2008b). *User Manual for the Injustice Experience Questionnaire.* Available online at: http://sullivan-painresearch.mcgill.ca/pdf/ieq/IEQManual.pdf (accessed August 26, 2018).

[B73] Sullivan M. J. Adams H. Horan S. Maher D. Boland D. Gross R. (2008). The role of perceived injustice in the experience of chronic pain and disability: scale development and validation. *J. Occup. Rehabil.* 18 249–261. 10.1007/s10926-008-9140-5 18536983

[B74] Sullivan M. J. Thibault P. Simmonds M. J. Milioto M. Cantin A. P. Velly A. M. (2009). Pain, perceived injustice and the persistence of post-traumatic stress symptoms during the course of rehabilitation for whiplash injuries. *Pain* 145 325–331. 10.1016/j.pain.2009.06.031 19643543

[B75] Sullivan M. J. L. Bishop S. P. Pivik J. (1995). The pain catastrophizing scale: development and validation. *Psychol. Assess.* 7 524–532. 10.1037/1040-3590.7.4.524

[B76] Tang N. K. (2008). Insomnia co-occurring with chronic pain: clinical features. Interaction, assessments and possible interventions. *Rev. Pain* 2 2–7. 10.1177/204946370800200102 PMC4589931 26525182

[B77] Thoresen S. Øverlien C. (2013). *Norwegian Translation of the Stressful Life Events Screening Questionnaire.* Oslo: The Norwegian Centre for Violence and Traumatic Stress Studies.

[B78] van Zuiden M. Heijnen C. J. Maas M. Amarouchi K. Vermetten E. Geuze E. (2012). Glucocorticoid sensitivity of leukocytes predicts PTSD, depressive and fatigue symptoms after military deployment: a prospective study. *Psychoneuroendocrinology* 37 1822–1836. 10.1016/j.psyneuen.2012.03.018 22503138

[B79] Verhey R. Chibanda D. Gibson L. Brakarsh J. Seedat S. (2018). Validation of the posttraumatic stress disorder checklist - 5 (PCL-5) in a primary care population with high HIV prevalence in Zimbabwe. *BMC Psychiatry* 18 109. 10.1186/s12888-018-1688-9 PMC5913864 29685117

[B80] Vlaeyen J. W. Linton S. J. (2000). Fear-avoidance and its consequences in chronic musculoskeletal pain: a state of the art. *Pain* 85 317–332. 10.1016/s0304-3959(99)00242-0 10781906

[B81] Waddell G. Newton M. Henderson I. Sommerville D. Main C. J. (1993). Fear avoidance beliefs questionnaire (FABQ) and the role of fear-avoidance beliefs in chronic low back pain and disability. *Pain* 52 157–168. 10.1016/0304-3959(93)90127-b 8455963

[B82] Weathers F. W. Litz B. T. Keane T. M. Palmieri P. A. Marx B. P. Schnurr P. P. (2013). *The PTSD Checklist for DSM-5 (PCL-5).*

[B83] Weiss D. S. Marmar C. R. (1997). “The Impact of Event Scale-Revised,” in *Assessing Psychological Trauma and PTSD*, eds Wilson J. P. Keane T. M. (New York, NY: Guilford Press).

[B84] Woby S. R. Watson P. J. Roach N. K. Urmston M. (2005). Coping strategy use: does it predict adjustment to chronic back pain after controlling for catastrophic thinking and self-efficacy for pain control? *J. Rehabil. Med.* 37 100–107. 10.1080/16501970410021535 15788345

[B85] World Health Organization (2018). *International Statistical Classification of Diseases and Related Health Problems (ICD-11).* Available online at: https://icd.who.int/browse11/l-m/en (accessed February 03, 2020).

